# Down-regulation of the Nucleotide Excision Repair gene *XPG *as a new mechanism of drug resistance in human and murine cancer cells

**DOI:** 10.1186/1476-4598-9-259

**Published:** 2010-09-24

**Authors:** Maria Antonietta Sabatino, Mirko Marabese, Monica Ganzinelli, Elisa Caiola, Cristina Geroni, Massimo Broggini

**Affiliations:** 1 Laboratory of Molecular Pharmacology, Istituto di Ricerche Farmacologiche "Mario Negri", 20156 Milan, Italy; 2 Nerviano Medical Science, Nerviano, Milan, Italy

## Abstract

**Background:**

Drug resistance is one of the major obstacles limiting the activity of anticancer agents. Activation of DNA repair mechanism often accounts for increase resistance to cancer chemotherapy.

**Results:**

We present evidence that nemorubicin, a doxorubicin derivative currently in clinical evaluation, acts through a mechanism of action different from classical anthracyclines, requiring an intact nucleotide excision repair (NER) system to exert its activity. Cells made resistant to nemorubicin show increased sensitivity to UV damage. We have analysed the mechanism of resistance and discovered a previously unknown mechanism resulting from methylation-dependent silencing of the XPG gene. Restoration of NER activity through XPG gene transfer or treatment with demethylating agents restored sensitivity to nemorubicin. Furthermore, we found that a significant proportion of ovarian tumors present methylation of the XPG promoter.

**Conclusions:**

Methylation of a NER gene, as described here, is a completely new mechanism of drug resistance and this is the first evidence that XPG gene expression can be influenced by an epigenetic mechanism. The reported methylation of XPG gene could be an important determinant of the response to platinum based therapy. In addition, the mechanism of resistance reported opens up the possibility of reverting the resistant phenotype using combinations with demethylating agents, molecules already employed in the clinical setting.

## Background

Drug resistance is one of the major obstacles limiting the effectiveness of cancer therapy [1-3]. Understanding the specific mechanisms of resistance to a given drug and the possibility of reversing the resistant phenotype are of pivotal importance.

It is generally accepted that DNA damaging agents show greater activity when there are defects in DNA repair. Exceptions are trabectedin, a marine compound currently under clinical investigation [4-7] that is less active in cells with deficient nucleotide excision repair (NER) [8,9] and cisplatin and carboplatin, two widely used anticancer agents which display resistance in cells lacking a functional mismatch repair (MMR) system [10,11]. MMR defects have been also reported to induce resistance to alkylating agents [12,13].

Nemorubicin is a 3'-deamino-3'[2-(S)-methoxy-4-morpholinyl] derivative of doxorubicin which has a 2-S-methoxymorpholinyl group at position 3' of the sugar moiety of doxorubicin. Nemorubicin is active *in vitro *as well as *in vivo *against murine and human tumor cell lines resistant to doxorubicin, to other P-glycoprotein and multidrug resistance protein (MRP) substrates and to topoisomerase II inhibitors [14-16]. It is also more potent than the parent drug and retains activity in tumors resistant to alkylating agents and topoisomerase I inhibitors. All these features strongly suggest that nemorubicin, although structurally an anthracycline derivative, has a completely different mechanism of action. Evidence that its activity can be enhanced by incubation with cytochrome P450 enzymes, particularly CYP3A, further differentiates its mechanism of action from classical anthracyclines [17]. The P450-dependent metabolism of nemorubicin, generates metabolites as active or more potent than the parent drug. Among these, 3'-deamino-3",4'-anhydro-[2"(S)-methoxy-3"(R)-hydroxy-4"-morpholinyl] doxorubicin (PNU-159682) has been isolated and synthesised; its potency *in vitro *is more than 1000 times that of the parent drug and it shows high antitumor activity *in vivo *with a spectrum of efficacy superimposable to that of nemorubicin [18]. Nemorubicin is under clinical evaluation for loco-regional therapy in hepatocellular carcinoma (HCC). In Phase I-II trials nemorubicin as single agent was effective against HCC patients; currently, phase I-II studies in combination with cisplatin are ongoing.

A murine cell line resistant to nemorubicin has been isolated and did not show cross-resistance to doxorubicin, topoisomerase I and II inhibitors, 5-FU, or vinblastine [19]. Interestingly, nemorubicin-resistant cells were hypersensitive to alkylating agents including melphalan, mitomycin C, platinum derivatives and nitrosoureas. All these characteristics prompted us to study the mechanism of action of nemorubicin in details, particularly the role of DNA repair mechanisms in its cytotoxicity.

## Results

**Figure 1:**
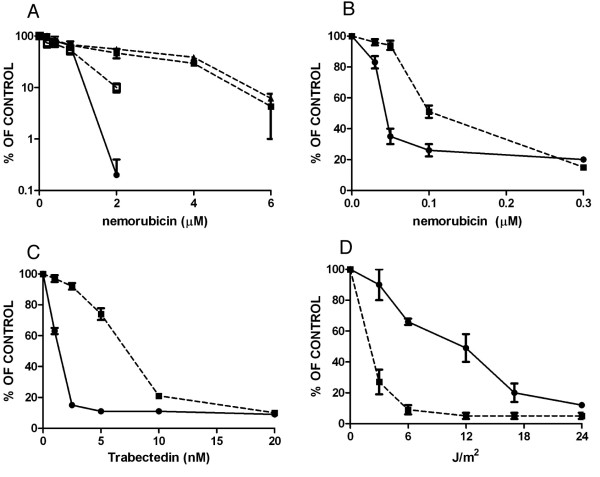
**Activity of nemorubicin in cells with NER defects**. Panel A. Inhibition of colony formation in CHO isogenic cells AA8 with a wt NER (black circle), CHO-UV96, black triangle) lacking the expression of ERCC1, CHO-UV61 (black square) lacking the expression of CSB/ERCC6 and ERA5 (white square) derived from UV96 in which the human ERCC1 gene was transfected and the NER activity restored. Results are reported as percentages of the inhibition relative to untreated cells and are the mean and SD of at least three replicates. Panel B. Activity of nemorubicin in L1210 cells with functional NER (L1210/DDP, black circle ) and NER defective L1210/0 (black square). Results are reported as percentages of the inhibition relative to untreated cells and are the mean and SD of at least three replicates. Panels C and D. Response of L1210 parental cells and of L1210 nemorubicin-resistant cells (L1210/MMDX) to UV (Panel D) and trabectedin (Panel C). Cells were treated with different concentrations of trabectedin or different UV doses and counted 72 hours later. The values are the mean +/- SD of three replicates. L1210 parental cells (black circle), L1210/MMDX cells (black square).

We tested the activity of nemorubicin *in vitro *in a CHO-derived system with defined NER defects (Figure 1A). Nemorubicin was less active in CHO-UV96 (lacking a functional ERCC1) and CHO-UV61 (defective in CSB/ERCC6) cells than parental AA8 cells. CHO-UV96 cells transfected with the human ERCC1 gene (ERA5) showed a restored NER function [8]; in this cellular system, sensitivity to nemorubicin greatly increased over CHO-UV96 deficient cells, approaching that found in parental CHO cells. A pair of isogenic murine leukemia cells were previously studied, L1210/0 (with defective NER function because of an XPG defect) and L1210/DDP (more UV-resistant because of restored NER function) [20]. We found that nemorubicin was more active in the L1210/DDP cells with intact NER than in the XPG-deficient L1210/0 cells (Figure 1B).

The effects on cells with defects in NER, were also tested for the potent nemorubicin metabolite [18], PNU-159682. The data reported in additional file 1 clearly show that the metabolite behaves as nemorubicin, being more active in cells with an intact NER. These effects have been found both in the CHO-derived clones and in the L1210 isogenic system used for nemorubicin.

We employed a murine L1210-derived cell line resistant to nemorubicin (L1210/MMDX) [19], and further characterised the sensitivity of parental and resistant cells to agents whose activity is influenced by NER. Nemorubicin-resistant cells were cross-resistant to the marine compound trabectedin, whose activity is NER-dependent [8,9], (Figure 1C) and the resistance index was similar to the one for nemorubicin. Treatment of these cells with UV light showed that nemorubicin resistant cells were four times more sensitive than parental cells to UV (Figure 1D).

**Figure 2:**
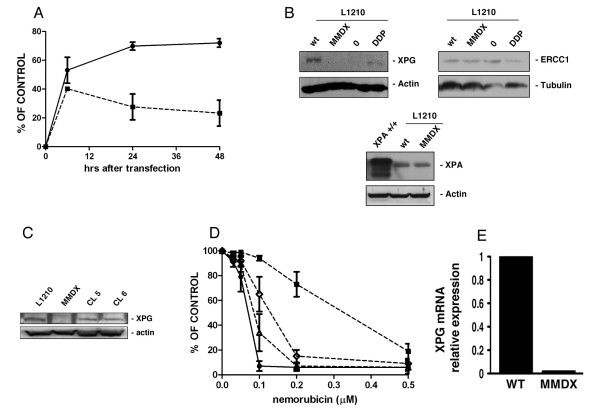
**NER Activity in sensitive and resistant L1210 cells.** Panel A. Host cell reactivation assay: L1210 parental cells (black circle), L1210/MMDX cells (black square). Panel B. Western blot analysis for the expression of NER-belonging genes ERCC1, XPA and XPG in parental (wt), nemorubicin resistant L1210 cells (MMDX), L1210/0 and L1210/DDP cells. Panel C. Western blot analysis for the expression of XPG in parental (L1210), nemorubicin resistant L1210 cells (MMDX), and in L1210/MMDX clone 5 (CL5) and clone 6 (CL6). Panel D. *In vitro *growth inhibition induced by nemorubicin in L1210 parental (black circle), L1210/MMDX (black square) cells and in two clones derived from L1210/MMDX (clone 5, (white triangle) clone 6 (white rhomb) ) in which the human XPG gene has been transfected. The values are the mean +/- SD of three replicates performed in three independent experiments. Panel E. Expression of XPG mRNA in L1210 parental and in L1210/MMDX cells measured by Real Time RT-PCR.

Using the host cell reactivation assay, we tested the NER-dependent ability of parental and nemorubicin-resistant L1210 cells to repair a damaged plasmid. Figure 2A shows that nemorubicin resistant cells were much less able to repair the lesions induced by UV than parental cells, indicating that NER impairment is likely in these cells.

We therefore analysed the expression of proteins involved in NER in parental and resistant cells and found that both L1210 nemorubicin-sensitive and resistant cells expressed comparable levels of ERCC1 and XPA (Figure 2B), while no XPG protein could be detected in resistant cells. L1210 nemorubicin-resistant cells were transfected with the human XPG cDNA and two independent clones re-expressing XPG were selected for testing the drug's activity. The two clones expressed the human XPG, as assessed by western blotting analysis (Figure 2C). The introduction of human XPG in L1210/MMDX cells was able to recover the compromised ability of these cells to repair UV-damaged plasmid (additional file 2). In both clones, restoration of XPG expression and function was associated with a restoration of nemorubicin activity, with an IC50 similar to the one in parental cells (Figure 2C).

Having shown that XPG defects are likely to be responsible for the resistance of these cells to nemorubicin, we analysed the molecular mechanisms responsible. A mutation in the XPG gene leading to premature stop codon was observed in a human cancer cell line made resistant to trabectedin [9]. We tested for mutations in the murine XPG gene of L1210 resistant to nemorubicin. Scanning the entire coding region of the gene and comparing the sequence with the one present in GeneBank, we did not find any mutations leading to a stop codon. By real time RT-PCR the mRNA levels of XPG in parental and resistant cells were analysed. The expression of XPG mRNA was negligible in the resistant cells (Figure 2C).

**Figure 3:**
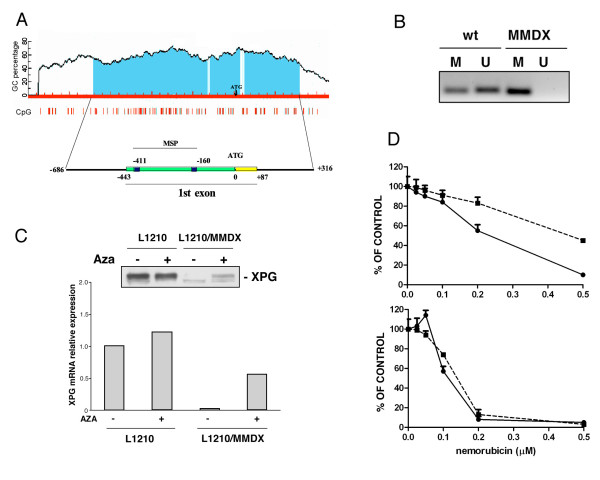
**Methylation analysis in L1210 murine model.** Panel A. Analysis of murine XPG promoter and identification of putative CpG islands from which primers for methylation specific PCR were designed. Panel B. Methylation specific PCR in L1210 sensitive (wt) and resistant cells (MMDX). M: methylated, U: unmethylated. Panel C. Expression of XPG mRNA and protein in L1210 parental and L1210/MMDX cells untreated or treated for 72 hours with 50 nM AZA. Panel D. Growth inhibitory activity of nemorubicin in L1210 parental (black circle) and L1210/MMDX (black square) cells without (up) or with (bottom) 50 nM AZA. Cells were pretreated with AZA for 72 hours before the addition of nemorubicin.

The lack of XPG mRNA expression prompted us to verify whether epigenetic mechanisms such as methylation of the promoter might account for the gene silencing. The murine XPG promoter contains a putative CpG island (Figure 3A) and primers were specifically designed to determine the methylation status of the promoter using methylation specific PCR. The results clearly indicate that the XPG promoter region analysed is methylated in nemorubicin-resistant cells (Figure 3B).

To further assess the importance of XPG methylation in determining resistance to nemorubicin, we analysed the expression of XPG mRNA and protein in L1210 parental and nemorubicin resistant cells treated with the demethylating agent 5'aza-deoxycytidine (AZA). This drug did not modify either the mRNA levels or the protein expression of XPG in parental L1210 cells (Figure 3C). In L1210-nemorubicin resistant cells, AZA partially induced the re-expression of XPG both at RNA and protein level. This increase paralleled the restoration of the sensitivity to nemorubicin (Figure 3D). Pretreatment with 5nM AZA for 72 hours alone induced in L1210 cells a reduction in growth and an increased activity when combined with nemorubicin. In L1210/MMDX cells, the pretreatment with AZA was able to revert the resistance to nemorubicin and the activity of the drug was similar to that observable in L1210 parental cells. Although the expression of XPG in L1210/MMDX cells treated with AZA did not reach the level present in L1210 parental cells, it was sufficient to repair UV-damaged plasmid with an efficiency similar to that of parental NER proficient cells (additional file 3).

**Figure 4:**
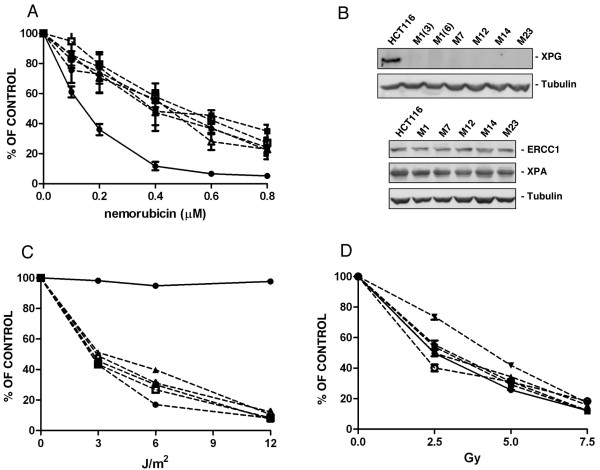
**Studies in parental and nemorubicin resistant human HCT116 cells.** Panel A. Inhibition of colony formation induced by nemorubicin in HCT116 parental cells (black circle) or resistant clones M1 (black square), M7 (white circle), M12 (black triangle), M14 (white square), M23 (white triangle). Results are reported as percentages of growth inhibition relative to untreated cells and are the mean and SD of at least three replicates. Panel B. Western blot analysis for the expression of NER-belonging genes ERCC1, XPA and XPG in parental and nemorubicin-resistant cells. Response of HCT116 parental cells (black circle) or resistant clones M1 (black square), M7 (white circle), M12 (black triangle), M14 (white square), M23 (white triangle) to UV treatment (Panel C) or γ-rays (Panel D). Results are reported as percentages of growth inhibition relative to untreated cells and are the mean and SD of at least three replicates.

**Figure 5:**
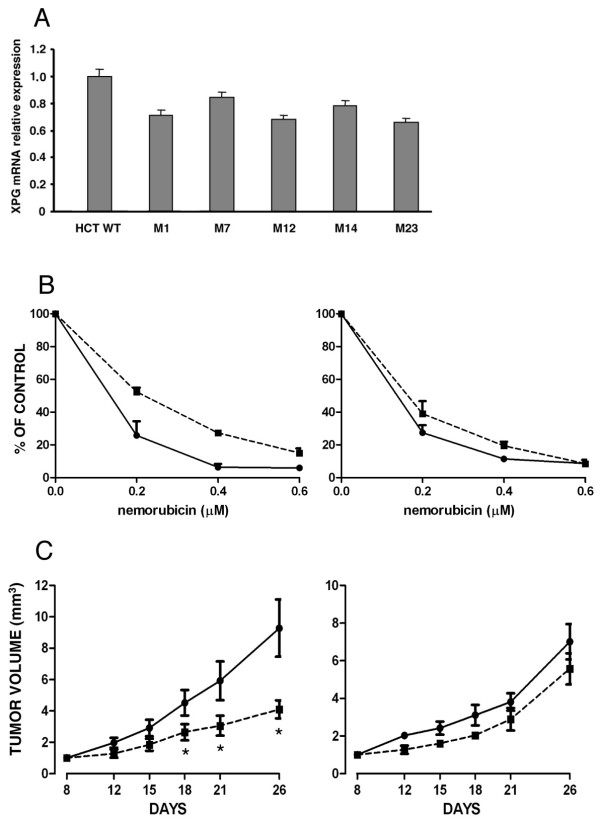
**In vitro and in vivo activity of nemorubicin in sensitive and resistant HCT116 cells.** Panel A. Expression of XPG mRNA in HCT116 parental and five nemorubicin-resistant cells measured by Real Time RT-PCR. Results are reported as decrease over untreated cells and are the mean and SD of at least three replicates. Panel B. *In vitro *cytotoxic activity of nemorubicin in parental HCT116 (black circle) and M23-resistant (black square) cells without (left) or with (right) 100 nM AZA. Panel C. *In vivo *activity of nemorubicin in mice transplanted with parental HCT116 (left) or the M23-resistant clone (right). Data are reported as tumor volume determined by measurement of the tumor diameters for untreated (black circle) or nemorubicin-treated (black square) mice. Each group consisted of 8 animals and each point represents the mean +/- SD. * p < 0.01 relative to untreated animals.

**Figure 6:**
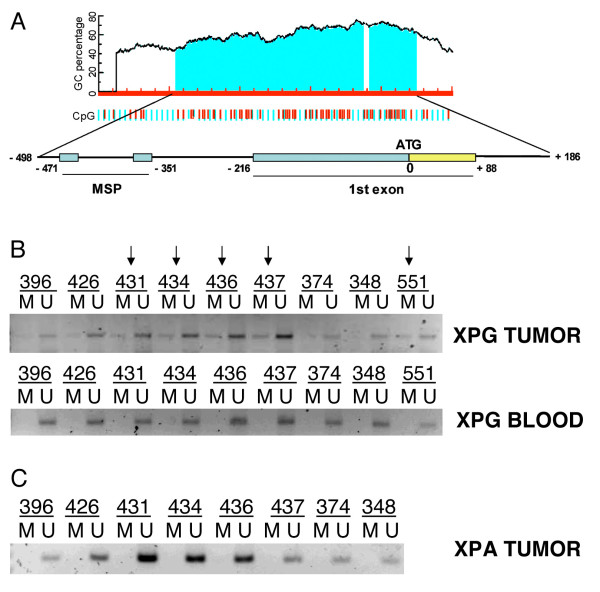
**Methylation of XPG promoter in human tumors**. Panel A. Analysis of human XPG promoter and identification of putative CpG islands from which primers for methylation specific PCR were designed. Panel B. XPG Methylation specific PCR in tumor and blood samples from patients with ovarian cancer. Samples in which methylated DNA was detectable have been highlighted. Panel C. XPA Methylation specific PCR in the same tumor samples used for XPG methylation analysis.

To select human-derived cancer cells for resistance to nemorubicin we isolated clones resistant to the drug from the human colocarcinoma cell line HCT116. We picked five independent clones which had a resistant index (from 2.3 to 3.1) similar to the one reported for murine cells (Figure 4A). Analysing the expression of NER genes in these clones, we found that all five resistant clones lacked XPG protein expression, but retained ERCC1 and XPA expression similar to parental cells (Figure 4B). The nemorubicin-resistant clones had increased sensitivity to UV rays (Figure 4C), but were equally susceptible to gamma rays (Figure 4D). The XPG gene was scanned and compared with the human XPG gene sequence present in GeneBank, and no mutations were found. HCT116 derived clones also displayed a 20-35% lower expression level of XPG mRNA, as detected by real time RT-PCR, than parental cells (Figure 5A). Analysis of the human XPG promoter revealed the presence of putative CpG islands (Figure 6A) which were analysed for methylation. In the regions selected methylation-specific PCR indicated no methylation (data not shown). Although we could not detect methylation in the HCT116 resistant clones despite a reduction in XPG mRNA levels, AZA treatment boosted the activity of nemorubicin in resistant clones but not in parental cells (Figure 5B), suggesting a small but appreciable role of methylation in this system as well. This same treatment with 5'aza-deoxycytidine, induced a very little re-appearance of XPG protein (additional file 4). One of the clones (M23) was selected for *in vivo *studies. Both sensitive and resistant cells grew at similar rate *in vivo*. M23 cells were found to be resistant to nemorubicin *in vivo *too (Figure 5C).

To verify whether the methylation of human XPG promoter could be detected in human samples too, we checked its status by methylation-specific PCR in 26 ovarian cancer DNA samples and the corresponding normal blood DNA. We found methylation in 5 out of the 26 tumor samples (approximately 20%), but not in blood DNA. Figure 6B reports a representative PCR result in these patients. Direct bisulfite sequencing confirmed the cytosine methylation in these samples (data not shown). Analysis of the XPA gene (Figure 6C) did not show any evidence of methylation beside the presence in its promoter of putative CpG islands.

## Discussion

Defects in DNA repair mechanisms are usually associated with greater sensitivity to anticancer agents [20-22]. Two major exceptions have been reported: defects in the MMR reduce the activity of cisplatin, carboplatin and alkylating agents, while defects in NER have been associated with a loss of susceptibility to treatment with the marine compound trabectedin, an interesting new drug currently under clinical investigation.

We have shown here that nemorubicin, a doxorubicin derivative currently in clinical evaluation, acts through a similar mechanism to trabectedin, requiring an intact NER system to exert its activity. Nemorubicin is an anthracycline derivative differing from doxorubicin for the presence of a 2-*S*-methoxymorpholinyl group in position 3' of the aminosugar. Doxorubicin has been reported to be more active in fibroblasts isolated from patients with defects in NER due to mutations in the XPD gene compared to human fibroblasts isolated from normal donors [23]. In the same isogenic system used for the experiments presented here, doxorubicin was found to be equally or only marginally more active in NER defective cells compared to wt, NER proficient cells (ratio of IC50 s in cells with NER defects and wt cells ranging from 1.2 to 1.4) [24]. The evidences reported here, together with the published lack of cross resistance with doxorubicin [19] make nemorubicin a compound clearly acting with a mechanism different from that of classical anthracyclines. The requirement of an intact NER system for nemorubicin activity has been demonstrated in murine and human cell lines. Furthermore we have found that cells, both murine and human, made resistant to nemorubicin show a defect in NER associated with the loss of expression of XPG. Cells resistant to nemorubicin are cross-resistant to trabectedin, although from a structural point of view, trabectedin and nemorubicin do not share similarities. Cell lines made resistant to trabectedin showed a multidrug-resistant phenotype [25,26] while nemorubicin did not induce this phenotype and cells resistant to doxorubicin through overexpression of MDR-1 retain sensitivity to nemorubicin [14,15]. Our findings indicate that nemorubicin, although structurally related to doxorubicin, acts with a different mechanism of action and this could influence the clinical development of the drug. In particular, our data show that at least in vitro, the resistance to nemorubicin involves XPG and is reversible. This could be an advantage in the clinic since there is the possibility to revert the methylation and possibly the resistance by demethylating treatments as reported for carboplatin [27].

As for the mechanism of inactivation of XPG found in nemorubicin-resistant cells, we did not find mutations in both human and murine XPG gene in resistant cells. The human cell line we made resistant to nemorubicin, the colocarcinoma derived HCT116, is the same human cancer cell line made resistant to trabectedin [9] for which a mutation in the XPG gene leading to premature stop codon was observed.

We have provided evidence that methylation of the XPG promoter is responsible for a lack of transcription of the gene in murine cells with resistance to nemorubicin. Promoter methylation is an important mechanism of gene silencing with a key role in cancer development where it can progressively reduce the expression of tumor suppressor genes favouring tumor initiation and progression [28-30]. In addition, an important example of methylation as a mechanism of induction of drug resistance is found in some cisplatin-resistant cells where the mismatch repair gene hMLH1 can be inactivated through this mechanism [31,32]. We herein report the first evidence of a methylation-dependent silencing of the NER belonging XPG gene. This mechanism is not restricted to a single experimental system, as it was observed in all the cells selected for resistance to nemorubicin. It is to note, however, that in the human colocarcinoma cell line HCT116 additional mechanisms responsible for XPG silencing are present. In fact, in these cells XPG protein expression is lost although mRNA expression can still be detected. These data, together with the lack of XPG methylation found in the DNA region analysed, would indicate that DNA methylation does not play a role in the XPG inactivation in these cells. However, the fact that pretreatment of nemorubicin-resistant HCT116 cells with 5'aza-deoxy-cytidine induces a small but appreciable increase in both activity and expression of XPG protein, would suggest that methylation could be present in CpG islands beside those analysed here. Clearly, the absence of XPG protein expression in the resistant clones would only partially be ascribable to this mechanism and post-trascriptional mechanisms not yet identified are more likely to play a role in these cells.

The data on XPG methylation were corroborated in clinical specimens where a substantial percentage of never treated ovarian cancers had low but detectable XPG methylation. In a small subset of patients, we could also measure the XPG mRNA levels. Although the number of samples analysed did not allow a proper statistical analysis, the results obtained in 5 XPG methylated and 9 XPG unmethylated samples showed that XPG mRNA levels in unmethylated samples were 1.5 fold higher than those in the methylated ones (data not shown).

The epigenetic-mediated induction of resistance opens up the possibility, as shown here *in vitro*, to revert the resistance phenotype using the drugs in combination with demethylating agents, already in clinical use. In addition, this mechanism of resistance has the advantage of inducing a particular sensitivity to DNA damaging agents such as platinum derivatives. The combination of nemorubicin and cisplatin is currently under clinical investigation and the first step of a phase II study in patients with hepatocellular carcinoma showed promising activity with good tolerability [33]. The evidence that XPG can be methylated in ovarian cancer samples, where platinum-containing regimens are used in first line, could help identify patients (with high XPG methylation) who are likely to benefit most from a platinum-based therapy, with a lower risk of relapse. This hypothesis is supported by the evidence that XPG expression has already been associated with response in ovarian and lung cancer [34,35]. Our cells lacking XPG are hypersensitive both to UV damage and cisplatin. The role of XPG methylation in determining response to platinum containing regimens needs to be tested in a larger cohort of patients with ovarian cancer.

## Materials and methods

### Chemicals

Nemorubicin hydrochloride and PNU-159682 were synthesized at Nerviano Medical Sciences (Nerviano, Italy). Trabectedin was kindly supplied by PharmaMar (Colmenar Viejo, Madrid, Spain). 5'aza-deoxycytidine was purchased from Sigma.

Stock solutions were prepared in water or DMSO and stored at -20°C

### Cells and drug-induced cytotoxicity

Chinese hamster ovary (CHO)-derived cells with specific defects in NER activity (UV96, UV61), kindly provided by Dr M Stefanini [24] were maintained in F10 medium supplemented with 10% FBS. Stably ERCC1-transfected CHO cells (ERA5, generated in our laboratory) were maintained in the same medium containing G418 500 μg/ml [8]. For clonogenic assays, cells were plated at 150 cells/ml and after 48 hours were treated for 1 hour with different drug concentrations. Colonies were stained with 1% crystal violet (Sigma-Aldrich) after 7-10 days and counted using the Entry Level Image analysis system (Immagini & Computer, Bareggio, Milan, Italy).

L1210 parental cells and L1210 cells resistant to nemorubicin [19], L1210/0 and L1210/DDP (kindly provided by Dr Wood [20]) were maintained in RPMI. Cells from each well were counted using a cell culture counter (Coulter Channelyser^® ^256, Beckman Coulter). Cells were seeded at 100.000 cells/ml and after 24 hours treated with different drug concentrations for 1 hour. The average number of cells from three wells determined 72 hours after treatment was used to assess growth rates. The mean ± SD is shown.

Human colocarcinoma cells HCT116 (obtained from American Type Culture Collection, Rockville, MD, USA), were maintained in ISCOVE's modified medium. Clones resistant to nemorubicin were obtained by culturing the cells in the constant presence of the drug. A clonogenic assay was used to determine drug activity. Parental HCT116 cells and five different clones (M1, M7, M12, M14, M23) were plated at 300-400 cells/well in six-well plates; 24 hours after seeding, different drug concentrations were added for 24 hours. The colonies formed were stained approximately two weeks later with 10% crystal violet in 20% ethanol.

IR treatments were made using Rad Gil Machine (Gilardoni - Italy) - UV treatment was performed using a UV lamp set at a dose of 100 J/sec.

Treatments with 5'aza-deoxycytidine were performed by adding the drug for 72 hours to L1210 and HCT116 cells at 50 and 100 nM, respectively. At the end of treatment, cells were harvested for molecular analysis or re-seeded for the determination of nemorubicin activity as described above.

### In vivo activity

Female athymic NCr-nu/nu mice (6 weeks old) were obtained from Harlan Italy (Udine, Italy). Mice were maintained under pathogen-free conditions and provided with food and water ad libitum. Procedures involving animals and their care are conducted in conformity with the institutional guidelines that are in compliance with national (D.L. n.116, G.U., suppl.40, 18 Febbraio 1992, Circolare No. 8, G.U., July 14, 1994) and international laws and policies (EEC Council Directive 86/609, OJ L 358,1, December 12, 1987; Guide for the Use of Laboratory Animals, U.S. National Research Council, 1996). 5 × 10^6 ^exponentially growing HCT116 cells (parental and M23 resistant clone) were injected per mouse subcutaneously. Nemorubicin was dissolved in sterile water and administrated i.v. at the dose of 0.05 mg/kg for each treatment (q7 d × 3) in a volume of 10 mL/kg body weight. The length (L) and the width (W) of the tumor mass were measured by caliper twice weekly and the tumor volume (TV) was calculated as TV = (L × W2)/2, being W < L. Differences in tumor growth between treated and untreated mice were compared by ANOVA analysis

### Host cell reactivation assay

Purified pGL2 plasmid was damaged with UV light (140 J). L1210 WT and L1210/MMDX cells were transfected with 5 μg of UV-treated pGL2 and 0.5 μg of untreated pRL-SV40 used for internal normalization using lipofectamine. Reporter gene activities were evaluated after 6, 24 and 48 h, using the Dual Luciferase system (Promega, Milan, Italy). Results are expressed as the percentage of the control luciferase reported activity normalized by the renilla activity value. The mean ± SD of three independent experiments is shown.

### Western blotting

For Western blot analysis, cells were lysed in ice-cold lysis buffer (50 mM tris pH8, 150 mM NaCl, 1 mM EGTA, 100 mM NaF, 10% glycerol, 1 mM MgCl2 and 1% Triton X-100) containing protease inhibitors (Sigma) and incubated on ice for 30 minutes. Samples were centrifuged at 13000 × g for 10 min at 4°C and the protein content of the supernatant was determined using a Bio-Rad Protein assay (Bio-Rad).

Forty μg of total cellular protein were resolved by SDS-PAGE on 8% polyacrylamide gel and electrotransferred to PVDF membrane. Immunoblot analysis was done using the following antibodies: anti XPG (Oncogene Research), anti XPA (FL-273) anti ERCC1 (FL-297) goat anti-actin and anti tubulin (H-235) (Santa Cruz Biotechnology). Membranes were then reacted with secondary antibodies (1:3000, Santa Cruz Biotechnology) and developed using the ECL kit (Amersham Biosciences).

### Real time RT PCR

Real Time RT-PCR was used for relative quantification of XPG mRNA. Three hundred ng of total RNA purified with the SV40 Total RNA Isolation System (Promega) were retrotransrcibed in 20 μl with an Archive Kit (Applied Biosystems) and 2 μl were amplified by Real Time PCR (ABI Prism 7900 Sequence Detection System, Applied Biosystems). XPG expression was detected using a commercial, TaqMan based, Assay by Design (M500164482, Applied Biosystems). Primers and TaqMan probe sequences to detect the actin mRNA levels were supplied as a ready-to-use solution (Applied Biosystems). Reactions were run in a total volume of 25 μL with TaqMan PCR Master Mix, following the manufacturer's instructions (Applied Biosystems).

### Mutation analysis

After genomic DNA extraction by using Maxwell 16 Cell DNA Purification Kit, exons amplification were performed by PCR with different couple of primers, for both human and murine XPG, listed in additional file 5.

PCR reactions were subjected to initial incubation at 95°C for 2 minutes, followed by 35 cycles of 95°C for 30 seconds, 54°C for 30 seconds and 72°C for 1 minute. Final extension was done by incubation at 72°C for 5 minutes. PCR products were separated on 1.5% agarose gels and visualized after ethidium bromide staining. Visualized bands were collected from gel, extracted and sequenced by using forward and reverse primers used for amplification. DNA sequencing were performed by PRIMM srl (Milan, Italy).

### Methylation specific- PCR

Genomic DNA was extracted for methylation analysis from cells in culture or frozen tissues by using Maxwell 16 Cell or Tissue DNA Purification Kit, respectively. One microgram of genomic DNA was modified with sodium bisulfite using the Epitect Bisulfite kit (Qiagen) according to the specifications of the manufacturer. Methylation-specific PCR (MSP) was run in a total volume of 25 μL by using AmpliTaq Gold (Applied Biosystems). MSP reactions were subjected to initial incubation at 95°C for 5 minutes, followed by 35 cycles of 95°C for 30 seconds, and annealing at the appropriate temperature for 30 seconds and 72°C for 30 seconds. Final extension was done by incubation at 72°C for 5 minutes. MSP products were separated on 2% agarose gels and visualized after ethidium bromide staining. To verify successful bisulfite modification of the DNA, a region of the Calponin promoter region was amplified with every modified DNA sample. The following primers were used:

#### Human XPG

Unmethylated Forward 5'-TTTGTGGATTTATTAGTGAAGGTGGG-3' Unmethylated Reverse 5'-ATAAAAACACATTAAAACAAAAAAAC-3' Methylated Forward 5'-GCGGATTTATTAGCGAAGGCGG-3' Methylated Reverse 5'-CACTAATAAAAACGCATTAAAACGAA-3'

#### Human XPA

Unmethylated Forward 5'-TAATTTGTGGAGTTTGTTTTG-3' Unmethylated Reverse 5'-CATTAACCATACCTCCAATAACCAC-3' Methylated Forward 5'-TCGCGGAGTTGTTTGTTTC-3' Methylated Reverse 5'-ATTAACCATACCTCCAATAA CC-3'.

#### Murine XPG

Unmethylated Forward 5'-AGGTATAGATTTAAAATTGAATTGT-3', Unmethylated Reverse 5'-CTCTTAACTTCCAAATAAACACAAA-3', Methylated Forward 5'-AGGTATAGA TTTAAAATCGAATCGT-3', Methylated Reverse 5'-CTTAACTTCCAAATAAACGCGAA-3'

DNA samples were obtained from 26 ovarian tumors (22 stage III and 4 stage I, as histotype: 18 serous, 3 endometroid, 2 mucinous, 1 clear cell, 1 undifferentiated, 1 germinal) derived form patients never treated with chemotherapy or radiotherapy. For these samples, together with methylation specific PCR, direct bisulfite sequencing was used to confirm the presence of methyl-cytosines.

## Competing interests

The authors declare that they have no competing interests.

## Authors' contributions

MB, MS and CG designed and coordinated the study. MS and EC performed the in vitro experiments on murine cell lines. MM performed sequencing and analysis of human cell lines, MG performed methylation in cells and human samples.

All authors read and approved the final manuscript.

## Supplementary Material

Additional file 1 Supplementary figure 1: Activity of PNU-159682 in cells with NER defects. Click here for file

Additional file 2 Supplementary figure 2: Host cell reactivation assay in sensitive and resistant cells. Click here for file

Additional file 3 Supplementary figure 3: Host cell reactivation assay in L1210 cells treated with AZA. Click here for file

Additional file 4 Supplementary figure 4: Western blot analysis of XPG expression in HCT116 parental and resistant cells. Click here for file

Additional file 5 Supplementary table 1: Sequence of primers used for amplification and sequencing of murine and human XPG gene. Click here for file
