# Supplementary figures and images for: Down-regulation of the Nucleotide Excision Repair gene XPG as a new mechanism of drug resistance in human and murine cancer cells
Source: Mol Cancer. 2010 Sep 24;9:259. doi: 10.1186/1476-4598-9-259 (PMC2955619; doi:10.1186/1476-4598-9-259)

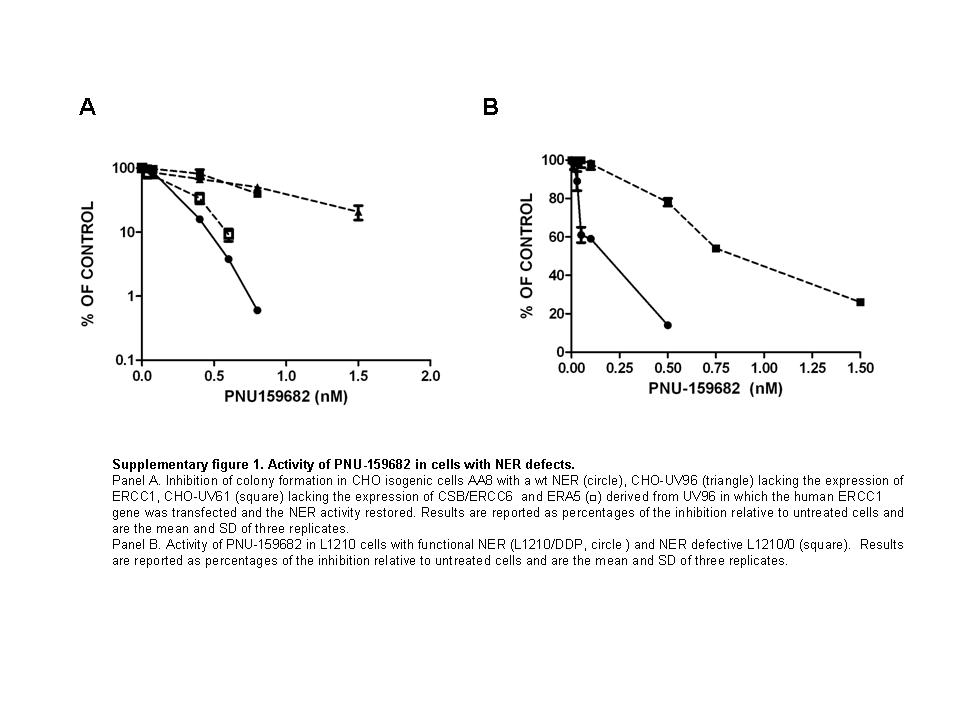

Supplement: Additional file 1 — Supplementary figure 1: Activity of PNU-159682 in cells with NER defects. [file 1476-4598-9-259-S1.JPEG]

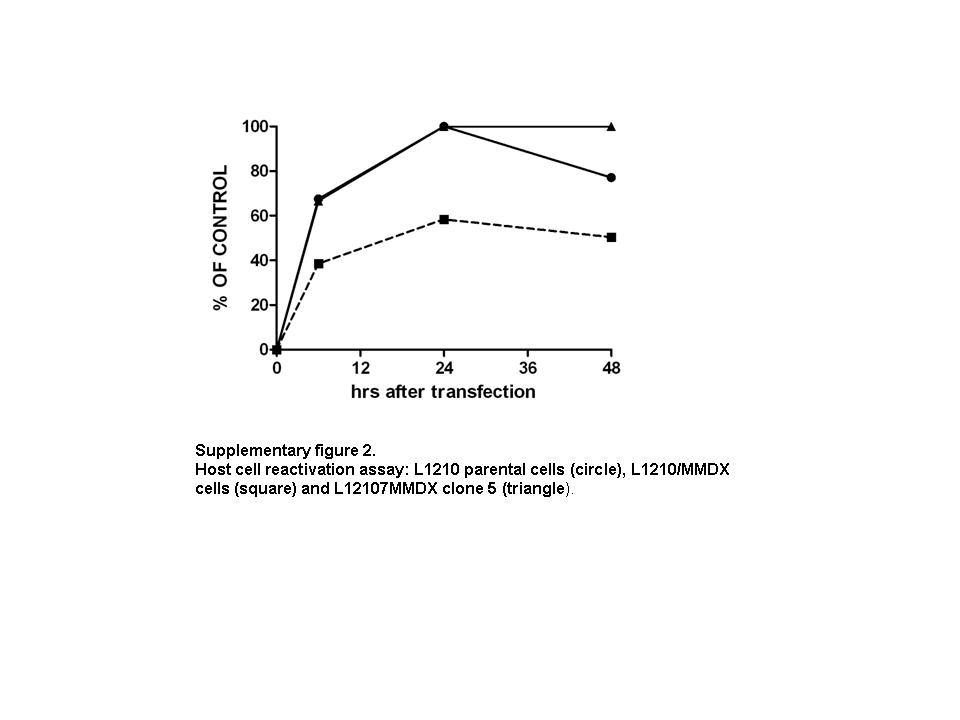

Supplement: Additional file 2 — Supplementary figure 2: Host cell reactivation assay in sensitive and resistant cells. [file 1476-4598-9-259-S2.JPEG]

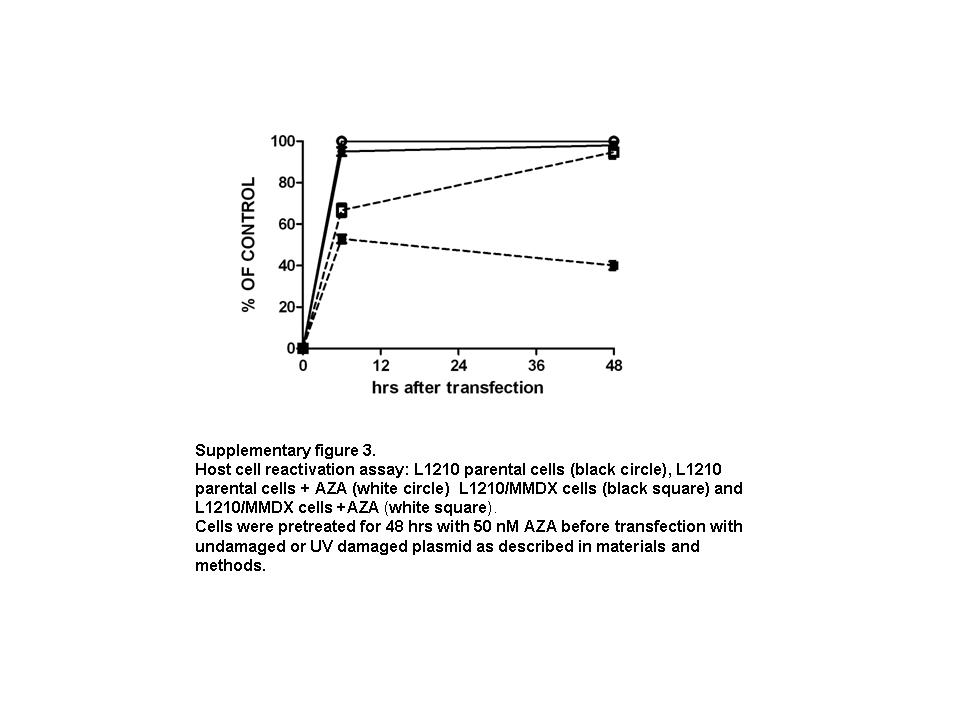

Supplement: Additional file 3 — Supplementary figure 3: Host cell reactivation assay in L1210 cells treated with AZA. [file 1476-4598-9-259-S3.JPEG]

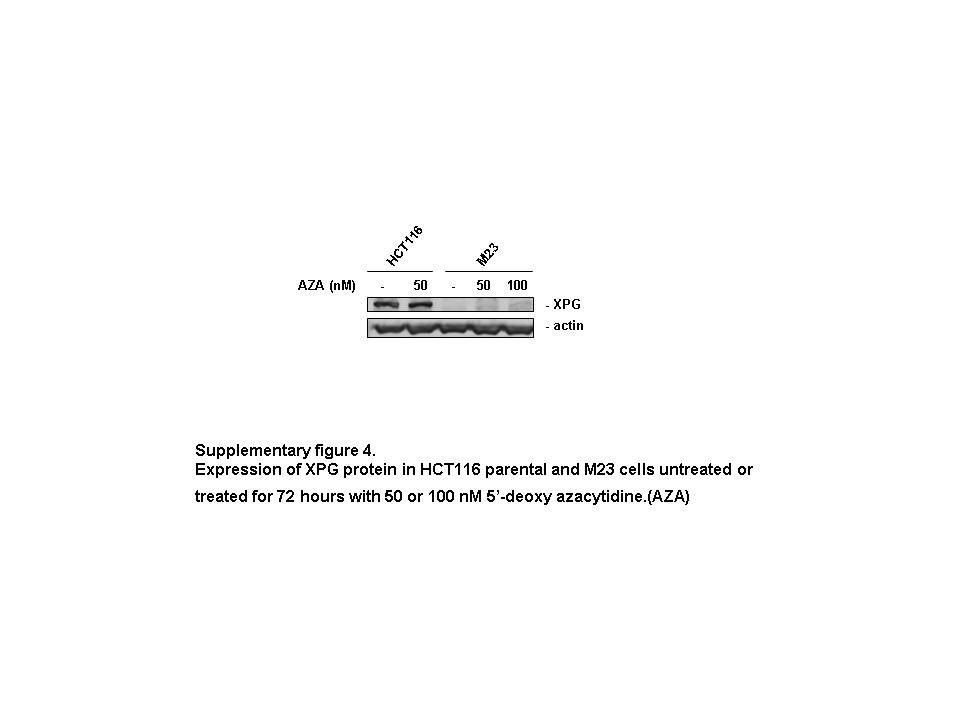

Supplement: Additional file 4 — Supplementary figure 4: Western blot analysis of XPG expression in HCT116 parental and resistant cells. [file 1476-4598-9-259-S4.JPEG]

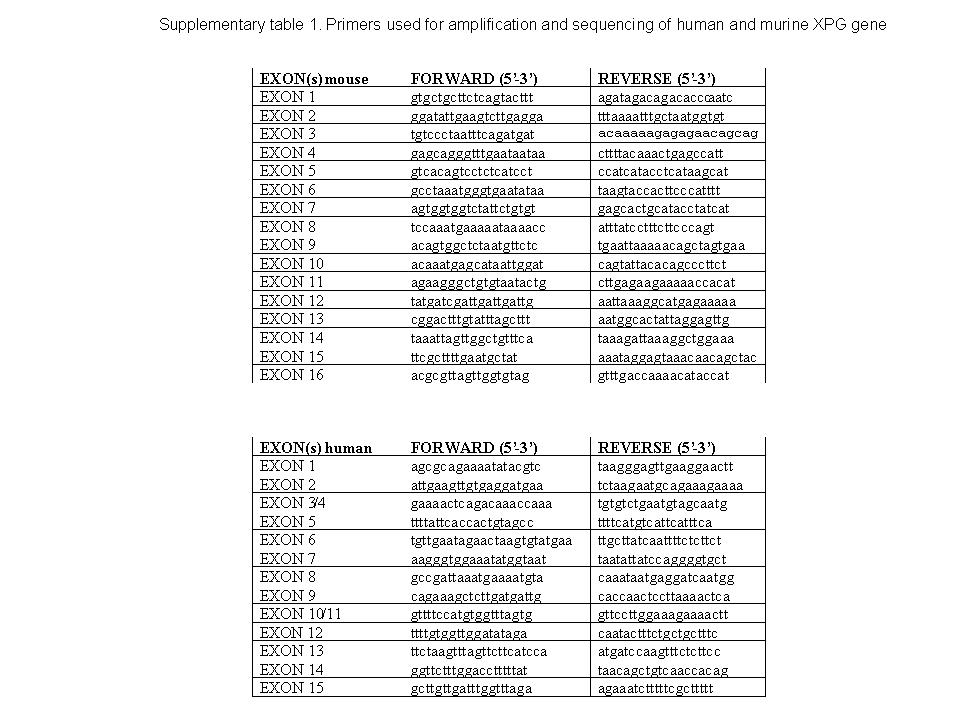

Supplement: Additional file 5 — Supplementary table 1: Sequence of primers used for amplification and sequencing of murine and human XPG gene. [file 1476-4598-9-259-S5.JPEG]
